# Professional quality of life among Norwegian oral health professionals working with torture and abuse survivors and patients with severe dental anxiety

**DOI:** 10.2340/aos.v85.45422

**Published:** 2026-01-28

**Authors:** Hayley Manalang Ko, Ingrid Volden Klepaker, Lubomiła Korzeniewska, Marte-Mari Uhlen-Strand

**Affiliations:** Oral Health Centre of Expertise in Eastern Norway, Oslo, Norway

**Keywords:** Oral health professionals, professional quality of life, burnout, secondary trauma, occupational well-being

## Abstract

**Objective:**

To investigate the level of burnout (BO), secondary traumatic stress (STS), and compassion satisfaction (CS) among Norwegian oral health professionals who work with traumatized and vulnerable patients.

**Material and methods:**

Professional Quality of Life-5 was used to assess BO, STS, and CS. Organizational and psychosocial work factors were measured with the Nordic Questionnaire for Psychological and Social Factors at Work.

**Results:**

Participants (*n* = 114) reported moderate levels across all three dimensions, with 62.6% scoring in the moderate range for STS, 47.4% for BO, and 38.6% for CS. Poor work-life balance was associated with lower levels of CS and higher levels of STS and BO. Support was associated with positive outcomes of BO, STS, and CS.

**Conclusions:**

Psychosocial factors within the work environment contribute more to occupational strain than the direct challenges of working with traumatized or vulnerable patients. Involving oral health professionals in wider conversations about supporting those who care for vulnerable populations is paramount. Addressing organizational conditions alongside individual support is important to promote well-being.

## Introduction

Mental health and wellbeing in oral health professionals are increasingly recognized [1]. Several studies have shown that dentists are among those healthcare providers particularly prone to burnout and mental health problems in their daily work [2–4]. Significant gaps remain in the literature, including how these issues affect clinical performance and patient safety [5]. Dental care relies on multifaceted expertise, such as clinical and technical competence, comprehensive and up-to-date knowledge in dental, oral and medical sciences, and the ability to build trust and communicate with patients. Despite the caregiving responsibilities for all population groups, including vulnerable populations, research on the professional quality of life of oral health professionals is virtually absent.

In Norway, the TADA (Torture, Abuse, and Dental Anxiety) service was established in 2011 as an interdisciplinary initiative to address dental avoidance among survivors of torture or abuse and individuals with severe dental anxiety. The service was developed to fill a gap in the national dental health service, recognizing that these groups often face substantial barriers to accessing routine dental care [6, 7]. The TADA service targets both psychological and oral health needs by integrating the expertise of clinical psychologists and oral health professionals to provide anxiety management and adapted dental care based on cognitive-behavioral therapy. Care is delivered within an interprofessional framework that ensures close collaboration between psychologists and oral health professionals. The program is delivered in two phases: phase one, involving dental and psychological staff, aims to reduce dental anxiety; phase two, led by the dental treatment team, provides individualized dental care tailored to the patient’s needs [8]. Onboarding packages, along with professional resources, guidelines, and manuals, are available on an internal website accessible to TADA professionals [8, 9].

The professional quality of life among individuals in caregiving professions has received increasing attention, reflecting a growing awareness of its implications for both the well-being of care providers and the quality of care delivered. A variety of terms, such as secondary traumatization, compassion fatigue, or vicarious trauma, have been used to describe the psychological challenges associated with caregiving, particularly the emotional distress resulting from exposure to narratives or accounts of trauma from those directly affected [10]. The compassion fatigue framework distinguishes three related but conceptually separate constructs [11]. *Secondary traumatic stress* (STS) is characterized by stress reactions arising from indirect exposure to others’ traumatic experiences and often presents with symptoms that closely resemble those of post-traumatic stress disorder (PTSD). *Burnout* encompasses the cumulative effects of a poor working environment and chronic occupational stress, typically manifested as emotional exhaustion and depersonalization [12]. In contrast, *compassion satisfaction* (CS) captures the sense of fulfillment derived from successfully helping others and performing one’s professional duties. Research has demonstrated that individuals working with people exposed to traumatic stressors are at risk of developing adverse symptoms such as burnout, compassion fatigue, depression, and PTSD. However, CS has been identified as a potential protective factor, mitigating the negative impacts of secondary exposure to trauma [11]. As oral health professionals in the TADA service work with traumatized individuals and operate in high stakes environments, these circumstances have the potential to cause trauma and stress that may affect their well-being and performance.

### Aim of the current study

The aim of the present study was to explore the professional quality of life among oral health professionals who work with torture and abuse survivors and patients with severe dental anxiety. The objectives were to describe the levels of burnout, STS, and CS, and examine how organizational and psychosocial factors were associated with these outcomes. It was expected that higher organizational demands and lower perceived support would be associated with higher burnout and STS, and with lower CS.

## Methods

### Participants

A total of 136 participants were recruited, of whom 22 were excluded due to failure to complete the study. Thus, the final sample included 114 TADA personnel (93.9% female) comprising dentists, dental hygienists, dental assistants/secretaries, dental specialists, and psychologists (*Table 1*). This aligns with the gender distribution in the Norwegian oral health workforce, in which women constitute a clear majority [13]. The sample therefore appears broadly representative of the current workforce composition.

**Table 1:** Descriptive characteristics of the study sample (n = 114).

Variables		*N*	%	Mean	*SD*
**Professional quality of life**					
**Compassion satisfaction**				39.8	4.79
	Low	30	26.3		
	Moderate	44	38.6		
	High	29	25.4		
**Burnout**				21.8	5.18
	Low	30	26.3		
	Moderate	54	47.4		
	High	30	26.3		
**Secondary traumatic stress**				20.6	5.49
	Low	26	22.8		
	Moderate	60	62.6		
	High	28	24.6		
**Step 1: Demographics**					
**Gender**	Female	107	93.9		
	Male	7	6.1		
**Age Group (years)**	< 30	8	7.0		
	30–40	36	31.6		
	41–50	28	24.6		
	51–60	35	30.7		
	> 60	7	6.1		
Country of education	Norway	103	90.4		
	Other Nordic	4	3.5		
	Non-Nordic	7	6.1		
Work experience (years)	< 10	26	22.8		
	≥10	88	77.2		
**Step 2: Work characteristics**					
**Profession**	Dental assistant	29	25.4		
	Dental hygienist	11	9.6		
	Dentist	53	46.5		
	Psychologist	18	15.8		
	Specialist	2	1.8		
	Administration	1	0.9		
**Workplace**	Public dental clinic	69	60.5		
	Private clinic	0	0		
	OHCE	36	31.6		
	University	1	0.9		
	Other	8	7.0		
**Employment status**	Permanent	107	93.9		
	Temporary	7	6.1		
**Clinic size**	<5	1	0.9		
	5–15	26	22.8		
	>15	87	76.3		
Patient days				4.14	1.27
Multiple employers	Yes	25	21.9		
	No	89	78.1		
**Management responsibilities**	Yes	14	12.3		
	No	100	87.7		
**Step 3: TADA experience**					
**Treatment team role**	Both	26	22.8		
	Exposure	86	75.4		
	Treatment	1	0.9		
	None	1	0.9		
**Years in TADA**	4 or less	57	50.0		
	5 to 8	36	31.6		
	9 or more	21	18.4		
**Step 4: Training and supervision**					
**Training program** ^1^	Yes	46	40.4		
	No	68	59.6		
**Course** ^2^	Yes	99	86.8		
	No	15	13.2		
**Voluntary work assistance** ^3^	Yes	89	78.1		
	No	25	21.9		
**Onboarding** ^4^	Yes	97	85.1		
	No	17	14.9		
**Step 5: Support and debriefing**					
**Desire for debriefing**				3.27	0.98
**Debriefing in workplace**	Yes	33	28.9		
	No	75	65.8		
	Don’t know	6	5.3		
**Frequency of debriefing per year**	Under 5 times	7	6.1		
	5–10 times	3	2.6		
	Over 10 times	25	21.9		
	Other	79	69.3		
**Support**	Supervisor			3.73	1.13
	Colleagues			4.39	0.724
	Friends/family			3.84	1.04
**Step 6: Psychosocial work factors**					
Quantitative demands				2.91	0.763
Decision demands				3.68	0.574
Learning demands				2.77	0.615
Role conflict				2.44	0.888
Role clarity				4.04	0.664
Work-life balance				2.54	1.07

OHCE: Oral Health Centre of Expertise.

1 Programs covering cognitive therapy and the clinical management of dental anxiety;

2 Theoretical seminars or courses lasting 1–3 days;

3 Peer consultations/supervision regarding cases;

4 Introductory training package for new employees, consisting of recorded lectures and digital meetings.

### Measures

The *Professional Quality of Life-5* (ProQOL-5; [11]) is a 30-item self-report measure that prompts participants to respond to questions about their feelings related to helping or caring for others in their work over the past 30 days. Based on the compassion fatigue framework [14], the instrument is composed of three subscales measuring CS, burnout (BO), and STS. Responses were recorded on a five-point Likert scale, ranging from 1 (never) to 5 (very often). Levels of CS, STS, and BO were divided into three categories based on the recommended cut scores: low (0–22), moderate (23–41), and high (42–50). Internal consistency was assessed using Cronbach’s alpha. The results indicated acceptable to good reliability: BO (α = 0.791), CS (α = 0.869), and STS (α = 0.824).

The *Nordic Questionnaire for Psychological and Social Factors at Work* (QPS Nordic) was used to measure organizational and psychosocial factors of work conditions [15]. The instrument was adapted for the current study due to its length; certain scales were omitted to create a more concise version. *Quantitative job demands* describe the amount of work to be completed within available time required. *Decision demands* capture the extent to which the job requires quick or complex decisions and sustained attention. *Learning demands* reflect the need for ongoing skill development and adaptation to new tasks. *Role clarity* concerns how well job responsibilities, goals, and expectations are understood, whereas *role conflict* refers to incompatible or competing demands. *Interaction between work and private life*, or work-life balance, reflects the degree to which work interferes with personal or family life.

The scales were scored on a five-point Likert scale, ranging from 1 (very seldom or never) to 5 (very often or always). Internal consistency was assessed using Cronbach’s alpha. Acceptable to good reliability was indicated for quantitative demands (α = 0.765), role clarity (α = 0.803), role conflict (α = 0.814), support from supervisor (α = 0.891), support from colleague (α = 0.832), and support from friends and relatives (α = 0.808). Decision demands (α = 0.599) and learning demands (α = 0.574) indicated poor reliability, falling below the threshold of α = 0.70. Thus, mean inter-item correlation (*MIC*) was calculated for decision demands (*MIC* = 0.344) and learning demands (*MIC* = 0.296). Both values fall within the acceptable range of 0.20 to 0.40 showing moderate internal consistency [16]. Given the theoretical validation of this scale in previous research, reliability estimates were deemed acceptable. Work-life balance was a single-item measure with a standard deviation of 1.07, suggesting good variability and reliability.

Two self-developed questions were included to assess participants’ perceptions of *emotional support*. The first question assessed participants’ feelings of support in the workplace, using a five-point Likert scale that ranged from 1 (to a very small extent) to 5 (to a very large extent). The second question aimed to identify the sources of their emotional support, such as family, friends, colleagues, supervisors, partners, and others.

Specific demographic characteristics pertinent to employment within the TADA service were assessed. Participants were also asked about the specific team they are part of and whether systematic debriefing processes are implemented in their workplace. Questions regarding participation in TADA-related professional development, including the completion of training courses or onboarding (an introductory training package for new employees) were included. For the ProQOL-5, an existing Norwegian translation that has been previously applied in research was utilized. The QPS Nordic scales were originally developed in Norwegian. For the ProQOL-5, an existing Norwegian translation that has been previously applied in research was utilized. The completion of the survey took 10 to 12 min.

### Procedure

An observational cross-sectional study design was utilized to explore the professional quality of life among professionals who work within the TADA service in Norway. Convenience sampling was chosen as the method for participant recruitment because participation depended on clinicians’ availability and willingness to complete the survey within their existing workloads. Participants were recruited in September 2024 at an annual conference for TADA professionals, where the study was presented, and the survey made available online via the TADA personnel resource page. Furthermore, the survey and reminder were distributed via the internal TADA newsletter system, independently of the research team. As the total number of employees working in the TADA service is not centrally documented, neither a formal response rate nor an approximate estimate can be calculated. Responses were stored in a secure database and anonymized to ensure that neither individual participants nor their affiliated agencies could be identified.

### Ethical approval

This study was granted approval by the Data Protection Officer of Østfold Municipality, ensuring compliance with relevant data protection regulations and ethical standards. Participation was voluntary and informed consent was obtained from the participants, who received no financial compensation.

### Statistical analysis

Due to extreme imbalance in gender and employment status, inferential statistical comparisons were not conducted because the sample (males, temporary) was too small to confirm normality and variance estimates would be unreliable. Independent samples t-tests, one-way ANOVAs, and Pearson correlations were employed for bivariate analysis. To ensure meaningful comparisons for profession type, one participant who worked in administration was excluded from this analysis. Hierarchical multiple regression analyses were performed to examine the unique contributions of predictors. Categorical variables were dummy coded. Predictor variables were categorized into six steps: demographics (Step 1), work characteristics (Step 2), TADA experience (Step 3), training and work assistance (Step 4), debriefing and support (Step 5), and psychosocial work factors (Step 6). After running the initial hierarchical regression, non-significant predictors (*p* ≥ 0.10) were removed, and only significant predictors (*p* < 0.10) were retained in the final model to improve interpretability [17]. Suppression effects were inferred when a predictor showed a non-significant or weak zero-order correlation with the outcome but became significant in regression with an increased beta coefficient, indicating that other variables controlled in the model suppressed irrelevant variance [18]. This was confirmed by examining predictor correlations and conducting additional regressions including suspected suppressors. Confounding was considered when a predictor was significant in bivariate analysis but lost significance after controlling for other variables suggesting shared variance. Data were analyzed using SPSS version 29 (IBM, Armonk, NY, USA).

## Results

### Compassion satisfaction

The average raw CS score of 39.8 (standard deviation [SD] = 4.79) reflected a moderate level of CS in the overall sample (*Table 1*). There was a significant difference in CS [*t*(112) = −2.31, *p* = 0.023], with those who have less than 10 years of work experience scoring lower than those who have more than 10 years of work experience. A one-way analysis of variance (ANOVA) was conducted to examine the differences between number of years worked in TADA. There was a significant difference between CS and years in TADA [*F*(2,111) = 4.51, *p* = 0.013]. Tukey's HSD (honestly significant difference) post hoc comparisons indicated that participants with 4 or fewer years reported significantly lower CS than those with 5 to 8 years (p = 0.041) and 9 or more years (p = 0.044).

When all predictor variables were included in the initial hierarchical regression, the full model explained 32.4% of the variance in CS [*F*(42,60) = 2.17, *p* = 0.003]. The simplified model explained 38.5% of the variance in CS, with a significant overall model fit [*F*(18,84) = 4.55, *p* < 0.001] (*Table 2*). Working in TADA for 5 to 8 years (β = 0.219, *p* = 0.015), receiving support from friends/family (β = 0.237, *p* = 0.012), and work-life balance (β = −0.396, *p* < 0.001) were significant predictors of CS. Individuals who had support from friends and family and worked for 5 to 8 years in the TADA service were more likely to report higher CS compared to those with 4 years or less experience. Workplace, specifically working at an oral health center of expertise (OHCE), was a significant predictor in the final model (β = −0.216, *p* = 0.019); however, no differences were observed in the bivariate analysis, suggesting classic suppression. When employment status was added, the effect of workplace strengthened, suggesting that employment status may have acted as a suppressor variable. Course participation predicted CS (β = −0.215, *p* = 0.016), despite not being associated with CS in bivariate analysis. No individual predictors were strongly correlated with both course participation and CS, making a clear classical suppression effect unlikely. A classical suppression effect was demonstrated with the significant predictor, decision demands. Learning demands (*r* = 0.383, *p* < 0.001) were found to be the suppressor variable. The initial relationship between work experience and CS may have been due to shared variance across multiple predictors.

**Table 2:** Hierarchical regression analysis predicting compassion satisfaction.

Predictors		1	2	3	4	5	6	*r*
Work experience		0.240*	0.286**	0.201	0.181	0.166	0.109	0.240
Workplace	Public clinic							
	OHCE		−0.150	−0.143	−0.150	−0.241*	−0.216*	−0.084
	University		−0.022	0.241*	0.015*	−0.004	−0.007	0.003
	Other		−0.115	−0.211	−0.186	−0.189	−0.116	−0.056
Employment status			0.202*	0.241	0.255	0.180*	0.163	0.113
Clinic size	< 5 employees							
	5–10 employees		−0.380	−0.214	−0.211	−0.581	−0.617	−0.040
	> 15 employees		−0.357	−0.263	−0.264	−0.627	−0.643	0.015
Role in TADA	Both							
	Exposure			−0.030	−0.038	−0.025	−0.027	−0.052
	Treatment			0.156	0.145	0.112	0.052	0.129
	None			−0.017	−0.026	−0.047	−0.076	−0.060
Years in TADA	4 or less							
	5 to 8			0.261*	0.277**	0.290**	0.219*	0.154
	9 or more			0.227*	0.220*	0.151	0.106	0.225
Course participation					−0.144	−0.166	−0.215*	−0.131
Supervisor support						0.203	0.114	0.245
Friends/family support						0.247*	0.237*	0.297
Decision demands							0.226*	0.032
Role clarity							0.098	0.347
Work-life balance							−0.396***	−0.431
*R* ^2^		0.058	0.120	0.218	0.229	0.349	0.494	
Adj. *R*^2^		0.048	0.056	0.114	0.117	0.235	0.385	
Δ*R*^2^		0.058*	0.063	0.098	0.011	0.118	0.147	
*F*-values		6.18*	1.86	2.09*	2.04*	3.08***	4.55***	

OHCE: Oral Health Centre of Expertise; TADA: Torture, Abuse, and Dental Anxiety.

Note. Standardized beta coefficients (β) are reported. Zero-order correlations (r) for the final model are reported.

* *p* < 0.05,

** *p* < 0.01,

*** *p* < 0.001.

### Burnout (BO)

The average raw score for BO was 21.8 (SD = 5.18) suggesting a low level of burnout (*Table 1*). There was a significant difference in BO [*t*(112) = −2.08, *p* = 0.04] between participants with multiple employers and those who only have one employer. A one-way ANOVA found a significant difference between professional group and BO [*F*(4,108) = 3.75, *p* = 0.007]. Due to unequal variances, post hoc comparisons using the Games-Howell test indicated that dentists reported significantly higher BO than psychologists (*p* < 0.001). Additionally, dental hygienists reported significantly lower BO than dentists (*p* = 0.009). A Pearson correlation indicated that individuals with higher BO were significantly more likely to indicate a need for debriefing (*r* = 0.346, *p* = 0.001).

Initially, the hierarchical regression model with all predictor variables had a total variance of 51.3% [*F*(42,60) = 3.56, *p* < 0.001]. The simplified model [*F*(12,90) = 12.57, *p* < 0.001] explained 57.6% of the total variance, demonstrating a better fit than the initial model (*Table 3*). In Step 2, profession significantly influenced BO and improved the overall model [Δ*R*^2^ = 0.156, Δ*F*(5,96) = 3.62, *p* = 0.005]. Profession and BO shared significant bivariate correlations with various work-related factors (e.g. decision and learning demands, need for debriefing, support, work-life balance). The addition of support in Step 4 was also significant [Δ*R*^2^ = 0.172, Δ*F*(1,91) = 28.1, *p* < 0.001] with supervisor support staying significant in the final model (β = −0.318, *p* < 0.001). Lastly, work-life balance was added (β = 0.500, *p* < 0.001), substantially improving the explanatory power of the model [Δ*R^2^* = 0.184, Δ*F*(1,90) = 44.2, *p* < 0.001]. Having one employer initially showed associations with higher levels of BO; however, this was not significant in the regression model. Correlational analysis showed that the initial association between BO and number of employers was confounded by role conflict. Similarly, bivariate analysis showed a significant positive correlation between BO and need for debriefing. which was confounded by learning demands, supervisor support, work-life balance, and profession (specifically, psychologists).

**Table 3:** Hierarchical regression analysis predicting burnout.

Predictors		1	2	3	4	5	r
Work experience		−0.143	−0.228*	−0.146	−0.127	−0.060	−0.143
Profession	Dental assistant						
	Dental hygienist		−0.142	−0.141	−0.148	−0.135	−0.135
	Dentist		0.216	0.301*	0.256*	0.066	0.298
	Psychologist		−0.218	−0.231*	−0.126	−0.115	−0.275
	Specialist		−0.007	0.030	−0.079	0.002	−0.031
	Other		−0.010	0.031	0.034	0.059	0.026
Role in TADA	Both						
	Exposure			0.200*	0.139	0.141	0.083
	Treatment			−0.134	−0.085	−0.062	−0.165
Years in TADA	4 or less						
	5 to 8			−0.107	−0.123	−0.045	−0.052
	9 or more			−0.194	−0.203*	−0.153	−0.172
Supervisor support					−0.439***	−0.318***	−0.519
Work-life balance						0.500***	0.663
*R* ^2^		0.021	0.176	0.271	0.443	0.626	
Adj. *R*^2^		0.011	0.125	0.191	0.375	0.576	
Δ*R*^2^		0.021	0.156	0.095	0.172	0.184	
*F*-values		2.12	3.42**	3.41***	6.57***	12.57***	

TADA: Torture, Abuse, and Dental Anxiety.

Note. Standardized beta coefficients (β) are reported. Zero-order correlations (*r*) for the final model are reported.

* *p* < 0.05,

** *p* < 0.01,

*** *p* < 0.001.

### Secondary traumatic stress

The average raw score for STS was 20.6 (SD = 5.49) indicating low levels of STS (*Table 1*). A significant difference in STS [*t*(112) = −2.36, *p* = 0.02] was indicated between participants with multiple employers and those who only have one employer. A significant difference in STS was also found between professions [*F*(4,108) = 3.28, *p* = 0.014]. Tukey HSD post hoc comparisons revealed that dentists experienced significantly higher levels of STS than psychologists (*p* = 0.005). There was a significant difference in reported STS levels and voluntary workplace assistance [t(112) = 2.14, p = 0.035]. Additionally, a significant difference was found between STS and onboarding [t(112) = −2.01, p = 0.047]. A Pearson correlation indicated that individuals with higher STS scores were significantly more likely to indicate a need for debriefing (*r* = 0.483, *p* < 0.001).

The initial hierarchical regression explained 53.4% of the total variance [*F*(42,60) = 3.79, *p* < 0.001]. After excluding non-significant variables, the simplified model improved [*F*(17,85) = 9.37, *p* < 0.001] explaining 58.3% of the total variance (*Table 4*). Significant predictors in the final model were need for debriefing (β = 0.219, *p* = 0.005), colleague support (β = −0.171, *p* = 0.031), and work-life balance (β = 0.494, *p* < 0.001). Colleague support and the desire for debriefing significantly improved the model [Δ*R*^2^ = 0.141, Δ*F*(2,85) = 11.92, *p* < 0.001]. Furthermore, the addition of decision demands and work-life balance improved the model significantly [Δ*R^2^* = 0.166, Δ*F*(2,85) = 20.3, *p* < 0.001]. However, decision demands (β = −0.238, *p* = 0.004) were not significantly associated with STS in bivariate analysis, suggesting a net suppression effect. Learning demands (*r* = 0.383, *p* < 0.001) were found to be the main suppressor, with work-life balance (*r* = 0.351, *p* < 0.001) likely acting as a secondary suppressor. Whilst bivariate analysis initially indicated an inverse relationship between onboarding and STS, regression analysis found a positive relationship (β = 0.186, *p* = 0.016). Supervisor support (*r* = −0.317, *p* = 0.001) was likely acting as a suppressor in the bivariate analysis. Similarly, working at an OHCE was a significant predictor in the final model (β = 0.257, *p* < 0.001); however, no differences were observed in bivariate analysis. Support from colleagues (*r* = 0.285, *p* = 0.002) and role conflict (*r* = −0.242, *p* = 0.009) were found to be suppressing the effect of workplace. Having one employer initially showed associations with higher levels of STS. Correlational analysis showed that the initial association between STS and number of employers was confounded by role conflict. Similarly, profession was initially significant in bivariate analysis. Profession and STS shared significant bivariate correlations with various work-related factors (e.g. quantitative and learning demands, role conflict, need for debriefing, support, work-life balance). Bivariate analysis initially showed that individuals who received voluntary work assistance reported significantly higher levels of STS. However, this relationship did not remain significant in the regression model. Furthermore, it did not share any correlated predictors with STS. Thus, the bivariate association may have been spurious and not robust once other variables were accounted for in the regression model.

**Table 4:** Hierarchical regression analysis predicting secondary traumatic stress.

Predictors		1	2	3	4	5	6	r
Work experience		−0.207*	−0.306**	−0.250*	−0.212*	−0.144	−0.107	−0.207
Profession	Dental assistant							
	Dental hygienist		−0.052	−0.028	0.017	0.001	0.045	−0.032
	Dentist		0.174	0.218	0.264*	0.243*	0.145	0.209
	Psychologist		−0.294**	−0.266*	−0.259*	−0.115	−0.129	−0.327
	Specialist		0.061	0.113	0.083	0.071	0.089	0.033
	Other		−0.088	−0.091	−0.077	−0.018	−0.004	−0.044
Workplace	Public clinic							
	OHCE		0.236*	0.240*	0.246**	0.295***	0.257***	0.163
	University		0.025	0.003	0.000	0.040	0.031	−0.106
	Other		0.051	0.051	0.027	0.021	−0.008	0.200
Multiple employers			0.167	0.177	0.179	0.157	0.113	0.200
Years in TADA	4 or less							
	5 to 8			−0.147	−0.233	−0.219*	−0.129	−0.101
	9 or more			−0.197	−0.214*	−0.085	−0.065	−0.138
Onboarding					0.227*	0.221*	0.186*	0.133
Need for debriefing						0.364***	0.219**	0.459
Colleague support						−0.254**	−0.171*	−0.226
Decision demands							−0.238**	0.081
Work-life balance							0.494***	0.655
*R* ^2^		0.043	0.270	0.304	0.346	0.486	0.652	
Adj. *R*^2^		0.034	0.191	0.211	0.250	0.393	0.583	
Δ*R*^2^		0.043	0.227	0.034	0.042	0.141	0.166	
*F*-values		4.54*	3.40***	3.28***	3.62***	5.49***	9.37***	

OHCE: Oral Health Centre of Expertise; TADA: Torture, Abuse, and Dental Anxiety.

Note. Standardized beta coefficients (β) are reported. Zero-order correlations (r) for the final model are reported.

* *p* < 0.05,

** *p* < 0.01,

*** *p* < 0.001.

## Discussion

The present study investigated the professional quality of life among oral health professionals working with highly traumatized patients, with a specific focus on burnout, STS, and CS. Overall, most participants reported moderate levels across all three dimensions of professional quality of life, with 38.6% scoring in the moderate range for CS, 47.4% for BO, 62.6% for STS. In contrast, Norwegian child protection service (CPS) workers have reported low levels of BO and STS [19, 20], whereas Norwegian therapists working in child mental health clinics have reported moderate to high levels of BO and STS [21]. This may suggest that oral health professionals in the TADA service are exposed to psychosocial stressors that mirror those encountered in other trauma-exposed professions. They could be impacted due to limited access to specialized resources for managing trauma-related challenges, in contrast to professions such as therapists and CPS workers, where there is a greater awareness of the inherently traumatic aspects of the work. Positive outcomes arising from secondary trauma, such as CS or secondary posttraumatic growth, could protect against BO and STS [22].

Psychosocial work characteristics consistently predicted outcomes across all three domains of professional quality of life. Poor work-life balance was associated with lower levels of CS and higher levels of both BO and STS. Alitabar and Parsakia [23] reported that work-life imbalance was a recurring theme in oral health professionals’ accounts of burnout, including descriptions of missed family events, difficulty maintaining personal relationships, and feelings of isolation due to demanding work schedules. Turnover intentions, and physical distress, which includes experiencing an adverse event related to patient safety, were found to have a negative effect on work-life balance, resulting in increased job stress [24]. However, Salloum et al. [25] found that creating a work-life balance plan, a core component of trauma-informed self-care, was not significantly associated with reduced STS. Yet, other studies point to the benefits of specific self-care strategies such as having social support from family, engaging in leisure activities, and taking time for rest—all which have been associated with lower levels of burnout and compassion fatigue [26, 27].

In the present study, higher decision demands predicted greater CS and lower STS, but this positive effect was weakened when learning demands were high and work-life balance was poor. Thus, working in an environment that supports autonomy may enable individuals to perceive stressful situations as challenges rather than stressors, allowing them to cope more effectively [28]. Conversely, insufficient decision-making authority, which can also increase role conflict, can exacerbate burnout and stress, with oral health professionals potentially experiencing frustration and helplessness, which can contribute to emotional exhaustion and reduced career satisfaction [23].

Support received outside the work environment was strongly associated with positive outcomes of BO, STS, and CS, highlighting its important role in coping with secondary trauma. Oral health professionals who relied on family members for emotional support also reported that it was instrumental in mitigating burnout [23]. Support from the work environment also had a protective role, with colleague support associated with decreased levels of STS and supervisor support with BO. The importance of a good psychosocial work environment has been reflected in the recent proposed amendments to the Norwegian Working Environment Act [29], which emphasizes the importance of organizational support as a crucial factor in mitigating perceived workload and psychosocial strain. It asserts that organizations should promote mental well-being at work, by addressing unclear or conflicting demands, emotional strain related to work involving close interaction with people, time pressure, and a lack of support.

Debriefing in work environments remained an underutilized practice, with 65.5% of respondents reporting the absence of debriefing opportunities. A desire for debriefing was associated with higher STS, likely reflecting efforts of individuals to manage their own stress, rather than the absence of debriefing being a direct cause of elevated STS [30]. The relationship between BO and the debriefing need was largely explained by other psychosocial work factors, consistent with Endsjø et al. [20] where work-related factors, rather than individual characteristics, consistently predicted CS, BO, and STS. Reported challenges and barriers to effective debriefing included inexperienced facilitators lacking knowledge of the relevant clinical context, difficulties in scheduling meetings, and the perceived intrusion on personal time [31]. Furthermore, the evidence regarding the effectiveness of debriefing as a strategy to prevent psychological distress is mixed with several systematic reviews (e.g. [32, 33]) questioning its efficacy and suggest it may sometimes worsen symptoms. Nevertheless, while debriefing may have perceived usefulness and potential benefits, organizational support remains a critical factor in mitigating workplace strain and distress [10].

Initially, onboarding was associated with lower levels of STS. However, after controlling for supervisor support, onboarding alone was found to be associated with increased STS within this sample. This may reflect that insufficient support within the broader work environment can diminish the beneficial impact of onboarding, resulting in new employees receiving little protection from stress. Furthermore, new employees may experience role ambiguity and uncertainty about organizational culture; but if these are not effectively managed, they may contribute to increased stress [34]. It is also possible that the effect observed partly captures the experiences of mentors responsible for onboarding, as the study did not differentiate between those providing and receiving onboarding. Acting as a mentor alongside existing workload demands may also impose additional strain, especially in work environments with low support [34]. Facilitating the transition of new professionals through structured and supportive onboarding is essential, rather than expecting them to adapt independently. This highlights that the quality and context of support, rather than the specific content or type of onboarding program, significantly influences outcomes [35].

TADA-related work characteristics were not robust predictors of STS, BO, and CS. The only notable finding was that individuals who had worked longer in TADA reported higher CS compared to those with less than 4 years of experience. This suggests that variations in professional quality of life are better explained by psychosocial work factors, rather than by exposure to indirect trauma or working with traumatized populations. Differences were also observed between workplaces, specifically between public dental clinics and OHCEs [36]. OHCEs have distinct responsibilities: they handle specialist cases, treat more complex conditions, and are responsible for educating dental specialists. While the complexity of TADA cases is generally consistent across both settings, the additional non-TADA cases managed at OHCEs may contribute to increased strain. This pattern may indicate that these experiences are more reflective of general workplace strain rather than secondary exposure to trauma.

### Limitations

Due to the cross-sectional design and absence of experimental manipulation or longitudinal data, causal inferences cannot be confidently drawn. Consequently, the findings should be interpreted as identifying associations and predictors rather than establishing definitive causal relationships. It is also possible that psychosocial factors and professional quality of life outcomes influence each other reciprocally, and the present design cannot determine the direction of these effects. Additionally, this study relied on convenience sampling and self-report measures which may limit representativeness and be influenced by social desirability bias and shape how participants portray their professional well-being, especially in contexts where expressions of vulnerability might be socially constrained. Furthermore, this study did not incorporate subjective indices of trauma exposure or personal history of trauma. Without this data, the potential moderating effects of personal history cannot be examined. The sample, drawn exclusively from the Norwegian dental service, may limit the generalizability of results to other settings. However, the issue of working with traumatized populations is relevant across oral health services internationally. Although the gender distribution reflects the composition of the current Norwegian oral health workforce, the predominance of women limits the possibility of meaningfully examining gender differences in professional well-being. Thus, interpretations should acknowledge this disparity as professional quality of life outcomes, stress responses, and help seeking patterns are known to vary by gender. While burnout and secondary trauma are consistently correlated and often co-occur, it is important to note the conceptual overlap between these constructs, especially within the context of the compassion fatigue framework [10].

Traditionally, predictors are selected for regressions based on strong bivariate correlations with the outcome, often excluding potential suppressors [37]. In contrast, this study initially included all variables, removing only those lacking significance in the regression analyses, to reduce selection bias. Final models were compared with bivariate results to explore meaningful partial relationships.

## Conclusions and implications

This study offers valuable insights into the professional quality of life experienced by oral health professionals working with traumatized populations, highlighting the multifaceted interplay between individual characteristics, occupational exposure, and psychosocial workplace factors. Future research incorporating trauma history could clarify whether certain individuals or subgroups are more susceptible to negative well-being outcomes. Furthermore, understanding how minority or underrepresented groups experience professional well-being remains an important area for future research. These findings highlight the critical need to include oral health professionals in the broader discourse surrounding support for those working with traumatized and vulnerable populations, ensuring that organizational psychosocial factors are addressed alongside individual-level factors to promote well-being.

## Data Availability

All data supporting the findings of this study are available from the corresponding author upon reasonable request.

## Geolocation information

This study was conducted across Norway, comprising a nationally representative sample of oral health professionals working in the TADA program (dentists, dental hygienists, assistants/secretaries, psychologists). The approximate geographic center of Norway is 63°N 12°E.
